# Supervision an essential tool in medical (and psychiatric) training

**DOI:** 10.1192/j.eurpsy.2024.1652

**Published:** 2024-08-27

**Authors:** R. J. Van Der Gaag

**Affiliations:** Psychiatry, Radboud University Medical Centre, Nijmegen, Netherlands

## Abstract

**Introduction:**

Supervision is an essentail tool in medical training and especially in psychiatry and psychotherapy. It emcopasses two distinct aspects namely: coaching in the workplace aimed at developping competences (pyramid of Miller) and on the other side a safe place to reflect on ones personal development as a professional. This mentoring should be distinguised from the personal training psychotherapy. The mentoring supervisor will take the doctor’s pledge as a starting point of professional development. But will also encourage the resident to become aware of elements of transference and counter transference in his/her clinical work, helping to foster empathy “maximal understanding with respect for professional distance. This, not only for the benefit of the patient and his/her security, but also in order to take care of one’s own health and developing a sound balance between work and private life.

Too often supervision is taken for granted once the resident has become a consultant. Along with examples of the techniques and the pitfalls of supervison, the presenters will plea for training and intervision as part of the development of the supervisor!

**Objectives:**

Raise awareness for the competences needed to become a valuable supervisor and the place supervision in its two aspects (coaching and place of reflection) should take in the training of medical doctors and especially psychiatrists

**Methods:**

- an inventory of the place of supervision in training in psychiatry throughout Europe
- looking into competences needed in order to develop a sound professional attitude
- looking into the competencies needed to become a valuable supervisor

**Results:**

- supervision is differently defined and given in training in psychiatry throughout Europe
- the competences needed in order to develop a sound professional attitude are defined in the Doctor’s Pledge (World Medical Association 2017) but should be refined according to the specialty with special attention to aspects of psychiatry alien to other specialities
- along with Teaching the Teachers - special trainings are available to become a supervisor

**Conclusions:**

Supervision is an essential tool in medical/psychiatric training, but it needs to be taken seriously in terms of developing the competences needed but also maintaining them in intervision with the colleagues of the training staff

**Disclosure of Interest:**

None Declared

